# Complete Mitochondrial Genome of the Human Liver Cancer HA22T Cell Line

**DOI:** 10.1128/genomeA.01136-17

**Published:** 2017-10-19

**Authors:** Deng-Gao Huang, Gang Cheng, Jin-Cai Wu

**Affiliations:** a Central Laboratory, Affiliated Haikou Hospital of Xiangya Medical College, Central South University (Haikou Municipal People’s Hospital), Haikou, Hainan Province, China; b Department of Radiation Oncology, Affiliated Haikou Hospital of Xiangya Medical College, Central South University (Haikou Municipal People’s Hospital), Haikou, Hainan Province, China; c Department of Hepatobiliary Surgery & Organ Transplantation, Hainan Provincial People’s Hospital, University of South China, Haikou, China

## Abstract

Liver cancer remains one of the most lethal malignancies worldwide. We sequenced the complete mitochondrial genome of a liver cancer HA22T cell line. The sequenced mitogenome of 16,603 bp will aid in the use of the HA22T cell line for liver cancer study.

## GENOME ANNOUNCEMENT

Liver cancer is one of the most lethal malignancies worldwide, causing 0.11 million deaths every year in China and accounting for 45% of all deaths in the world (1). As this disease is lethal in most cases, research has to be done to improve our understanding of the disease and to offer insights for possible treatment options. HA22T is a perpetual cell line which was derived from the liver tissue of liver cancer with a well-differentiated hepatocellular carcinoma. These cells are epithelial in morphology, and are not tumorigenic in nude mice. The cells secrete a variety of major plasma proteins, e.g., albumin, transferrin, and the acute-phase proteins fibrinogen, alpha 2-macroglobulin, alpha 1-antitrypsin, transferrin, and plasminogen. They have been grown successfully in large-scale cultivation systems. Hepatitis B virus surface antigens have not been detected. HepG2 will respond to stimulation with human growth hormone. The immortalized human liver cells constitute an *in vitro* model for pharmacotoxicological studies and for the investigation of the etiology and pathogenesis of human liver cancer (1).

Mitochondrial DNA (mtDNA) is a closed double-stranded circular molecule that typically contains 37 genes, including two rRNAs and 22 tRNAs (2). Previous studies (2, 3) have suggested that mitochondrial DNA instability and variation are associated with many diseases in both animals and humans. Thus, sequencing the complete mitochondrial DNA sequence would aid in the clinical study of the disease and therapy.

In this work, we report the complete mitochondrial genome sequence of the liver cancer HA22T cell line. The total length of the complete mitogenome of the HA22T cell line is 16,603 bp. The whole mitochondrial genome contains 22 tRNA genes, two rRNA genes, 13 protein-coding genes, and a control region (D-loop region). The overall G+C content of the mitogenome was estimated to be 39.2%, for A (34.9%) > T (26.1%) > C (26.0%) > G (13.0%). The total length of 13 protein-coding genes is 11,404 bp. With the exception of *ND2*, *ND3*, and *ND5* that use the start codon ATA and *ND4L* that uses GTG, the other protein-coding genes use ATG as the start codon. When it came to stop codons, six genes terminated with TAA, whereas *ND1* and *ND2* terminated with TAG and *Cytb* terminated with AGA. In addition, four genes terminated with incomplete stop codon T- (*COX2*, *COX3*, *ND3*, and *ND4*), which presumptively formed a complete stop codon by posttranscriptional polyadenylation. The 22 tRNA genes ranged in size from 59 bp (*tRNA*^*Ser*^) to 77 bp (*tRNA*^*Leu*^). The control region of the mitochondrial DNA is located between the *tRNA*^*Pro*^ and *tRNA*^*Phe*^ genes and measures 1,019 bp. The small noncoding region, a putative origin of the light-strand replication, was located between *tRNA*^*Asn*^ and *tRNA*^*Cys*^ genes with a length of 41 bp. This mitochondrial genome sequence will aid in the use of the HA22T cell line for liver cancer study.

### Accession number(s).

This mitochondrial genome sequence has been deposited at DDBJ/ENA/GenBank under accession no. MF716582. The version described in this paper is version MF716582.1.
